# Preventing suicide in refugees and asylum seekers: a rapid literature review examining the role of suicide prevention training for health and support staff

**DOI:** 10.1186/s13033-022-00534-x

**Published:** 2022-05-14

**Authors:** Jessica Ingram, Bronte Lyford, Amanda McAtamney, Sally Fitzpatrick

**Affiliations:** 1 Everymind, 72 Watt St, Newcastle, NSW 2300 Australia; 2 School of Medicine and Public Health, University of Newcastle, College of Health, Medicine and Wellbeing, NSW 2308 Callaghan, Australia

**Keywords:** Refugee, Asylum seeker, Suicide, Training, Suicide prevention

## Abstract

**Background:**

Refugees and asylum seekers are exposed to a unique set of circumstances and experiences that are associated with an increased suicide risk. Suicide prevention training has been recognised as a central component supporting a comprehensive approach to suicide prevention. Limited literature exists exploring the role of suicide prevention training for health and support staff working with refugee and asylum seeker consumers.

**Methods:**

To determine the impact suicide prevention training for health staff may have in supporting refugee and asylum seeker suicide prevention, researchers undertook a rapid literature review exploring what elements should be considered when developing suicide prevention training for health and support staff working with refugee and asylum seeker consumers.

**Results:**

Results of academic and grey literature screening identified 14 studies exploring suicide prevention training for health and support staff working with refugee and asylum seeker consumers. Findings of the literature review suggest suicide prevention training for health and support staff working with refugee and asylum seekers should consider the inclusion of content which increases participant competence and confidence to identify and respond to suicide risk; provide staff with an understanding of cultural differences and its impact on refugees and asylum seekers recognition of mental health and suicide as a health matter; highlight the importance trauma informed practices in care and consider the lived experience of refugees and asylum seekers.

**Conclusions:**

Inclusion of specific content in refugee and asylum seeker suicide prevention training may provide health and support staff increased competence and confidence to identify and respond to suicide risk in refugees and asylum seekers.

## Introduction/Background

The United Nations High Commissioner for Refugees estimates approximately 79.5 million people are forcibly displaced worldwide [1]. Refugees and asylum seekers are exposed to a unique set of circumstances and experiences throughout their journey and re-settlement, which may contribute to trauma and distress [2]. The complexity of political, social and economic contexts, combined with cultural factors and individual biological and physiological factors may result in an increased rate of psychological distress and suicide in refugees and asylum seekers [2, 3].

Suicide is the act of deliberately ending one's life, although it can be difficult to determine if a person intended their actions to result in death. Suicidal ideation refers to a person having thoughts of ending their own life. These thoughts may vary in intensity and duration from fleeting thoughts to a complete preoccupation with wanting to die. Although not all suicidal thoughts lead a person to suicide or attempt suicide, suicidal ideation should always be taken seriously [4, 5]. Suicide risk refers to the full spectrum covering suicidal ideation, through to suicide attempts and an individual dying by suicide. There is no single factor that contributes to suicide or suicidal behaviour. It is important to acknowledge the complexity and interaction between many risk and protective factors for refugees and people seeking asylum.

Research in the field of suicidality suggests that suicide is a largely unpredictable outcome that arises from a complex interaction between many vulnerabilities and risk factors in a person’s life [6]. Refugees and asylum seekers are exposed to many vulnerabilities from their experiences. These experiences, in conjunction with uncertainty for their future resettlement and safety, place refugees and asylum seekers at a heightened risk of suicide. Some of the core factors found to contribute to suicide risk in refugees and asylum seekers include temporary visa status, exposure to trauma, exposure to detention settings, and social isolation. Proctor et al. [7] coined the term ‘lethal hopelessness’ to describe the increased suicide risk in asylum seekers due to the combination of limited access to mainstream services, financial support, culturally safe health care, and working rights. These authors suggest that a public health approach to reducing distress and suicidality should address distress in asylum seekers and the availability of immediate support from mental health professionals to assist with the successful re-settlement of asylum seekers [7].

### Visa status and suicide risk

Refugees and asylum seekers experience long periods of displacement and insecurity due to the often-lengthy visa process. Nickerson et al. [8] found that refugees who are granted temporary visas experience a great deal of uncertainty contributing to negative mental health outcomes whilst their visas are processed. Refugees living on temporary visas experience higher levels of suicidality, depression and post-traumatic stress disorder (PTSD) symptoms than those on secure or permanent visas [8, 9].

### Trauma

Exposure to trauma is a known factor linked to suicidality [10]. A study by Bojic et al. [17] exploring the long-term mental health of war refugees exposed to high levels of trauma, found that prevalence of mental illness including depression, PTSD and anxiety disorders were in the range of 20% or above for refugees post re-settlement. Trauma informed interventions, with particular focus on supporting previous trauma and an individual’s migration experience, whilst also including content to support migration into the resettlement country may be most effective in reducing psychological stress and suicide [7].

### Detention settings

Asylum seekers held in detention settings are placed at an increased risk of psychological distress and suicide [11, 12]. Conditions experienced by asylum seekers within detention settings may place asylum seekers in a position of isolation, with increased exposure to trauma, exposure to physical and sexual abuse, and limited rights, contributing to suicide risk [13]. An analysis of self-harm in asylum seekers by Dudley [14] found that suicidal behaviour in individuals living in detention was much higher than rates of suicidal behaviour of the comparison general population [14].

### Social isolation, loss and re-settlement

Refugees and asylum seekers are placed at an increased risk of experiencing feelings of isolation if they are separated from their families, culture and spirituality [8], 15]. Loss of culture and connection through resettlement may be detrimental to the mental health of refugees and asylum seekers [12]. Although refugee and asylum seeker pre-migration experiences highly influence mental health and wellbeing, studies suggest that refugee and asylum seeker mental health is also shaped by life experiences post-migration [9], with stable resettlement processes supporting positive mental health outcomes [12].

### Social determinants and mental health and suicide risk of refugees and asylum seekers

Refugees and asylum seekers may be vulnerable to poor physical and mental health due to the inequalities stemming from the social determinants of health [16]. On arrival to a different country, refugees and asylum seekers may be at an increased exposure to poor social conditions, where they may live at a low end of the social gradient [9, 16]. Bojic et al. [17] found that poor socioeconomic status, like that experienced by refugees and asylum seekers is known to be associated with depression and anxiety [17].

Poor financial situations coupled with visa restrictions limiting access to services, welfare and health care in the country of resettlement place refugees and asylum seekers in a vulnerable position impacting both mental and physical health [9]. Visa restrictions may limit opportunities for refugees and asylum seekers to generate income through employment which impact food and housing security, as well as social support and connectedness if affordable housing is not located in supportive, well serviced communities [9].

### Refugee and asylum seeker suicide prevention training as an early intervention approach to suicide

Existing evidence suggests mental illness and suicide in refugees and asylum seekers may be prevented through early intervention practices [18, 19]. Suicide prevention training as an early intervention approach may improve ability of training participants to understand, identify and manage suicidality in persons experiencing suicidal ideation or psychological distress [20–22]. Within other population groups, suicide prevention gatekeeper training has been recognised as an integral part of a comprehensive approach to suicide prevention [21, 23, 24]. Suicidality may be reduced in individuals experiencing distress if they are supported by someone trained in specialist suicide prevention intervention skills [25].

Health workers and support staff who work with refugees and asylum seekers are in a unique position to identify signs of mental illness and suicidality among refuges and asylum seekers because of the close proximity of which they work. The development of refugee specific suicide prevention training that is delivered to health and support staff working with refugee and asylum seeker consumers may support an early intervention strategy to suicide prevention. Limited research exists about the effectiveness of suicide prevention training to improve ability for health and support staff working with refugees and asylum seekers to effectively identity and manage suicide risk in refugee and asylum seeker clients.

Given the increased risk of suicide for refugees and asylum seekers, and the limited research exploring the role of suicide prevention training to support an early intervention approach to suicide prevention in refugees and asylum seekers, this rapid literature review aims to explore existing suicide prevention training content for health and support staff working with refugees and asylum seekers.

## Methodology

### Eligibility criteria

#### Inclusion criteria

To be eligible for inclusion in the rapid literature review, publications were limited to full text articles in English only, were published between January 2000 and January 2022, were journal articles, books, reports, reviews, or official publications, and the sources of literature for inclusion were from academic journals, government and official publications, reports, grey literature. The date of the literature search was February 2022.

#### Exclusion criteria

I. Literature that was not in English, full text was not accessible
II. Literature that was not published between January 2000 and January 2022
III. Literature that was not classified as a journal article, book, report, review, official publication, or literature review
IV. Literature that did not focus on mental health or suicide prevention training or refugee health practices in the context of health and support staff working with refugees and asylum seekers.

### Information sources and search strategy

Due to limited existing research in suicide prevention training for support staff working with refugees, search terms remained broad to ensure that all relevant publications were captured in the search. The search terms included key words for suicide prevention and mental health, asylum seeker, and refugee, which were combined using the Boolean Operator “AND” with search terms suicide, prevention, training, health, staff, worker, employee, refugee, and asylum. An example search strategy and search terms are referenced in Appendix 2 and 3.

### Academic literature

A literature search specialist from Hunter New England Health Libraries (New South Wales, Australia) was consulted to identify key databases suitable for the literature search. The following databases were searched to increase the likelihood of capturing relevant publications given the recognition of limited research in the space of refugee and asylum seeker health. Databases included BioMed Central (Part of Springer Nature), EBSCOhost Academic Search Ultimate, EBSCOhost- Sociology Source Ultimate, ELSEVIER Clinical Key, ELSEVIER Science Direct, Informit Health Collection, ProQuest, PubMed Central, SAGE journals, Taylor & Francis Online, and Wiley Online Library were selected for the literature search.

To increase identification of relevant published and grey literature, authors also searched publication lists of known prominent researchers working in the refugee and asylum seeker suicide prevention field.

### Grey literature

Grey literature search focused on data and statistics published by Australian government departments and leading refugee and asylum seeker organisations in Australia and internationally.

### Literature selection

Search results were exported into Endnote X6. Duplications were removed from the search results. Two authors identified and screened literature results for eligibility for inclusion in the review according to eligibility criteria, with literature selection undertaken by reading the title and abstract of each literature source. Any discrepancies regarding suitability for inclusion in the rapid literature review that arose between the two authors were reviewed and resolved by the third author.

The literature results remaining after screening procedures and inclusion criteria were applied were read in full by two authors to identify key themes, information and content that relate to the aim of the rapid literature review.

### Screening

To narrow the results of the database and grey literature search, all publications were screened against title and abstract in reference to inclusion and exclusion criteria and the focus questions of the rapid literature review. Further full text reading was undertaken for papers thought suitable from the title and abstract screening stage. Eligible papers were saved within EndNoteX6 software.

A total of 14 publications were deemed eligible (n= 378 excluded), of which are referenced in this rapid literature review.

### Data extraction

Of the final 14 results of the literature search, one author read through the full text papers and identified content relating specifically to (i) elements of suicide prevention training including content and format, (ii) type of staff undertaking the training, and iii) cultural sensitivities relating to suicide prevention training. Extracted data was categorised under key themes and topic areas. These topic areas were distinguished during the analysis of full text literature included in this review. To inform the results, two authors identified and developed key subheadings that would be best suited to logically present the findings of the rapid literature review for each topic area. Table 1: *Study characteristics* presents the final literature accepted for the review

**Table 1:** Study characteristics

	Theme	Year	Author	Title	Country of publication	Type	Description
1	Suicide prevention training for staff supporting refugees and asylum seekers	2021	Löfving Gupta	Readiness of Allied Professionals to Join the Mental Health Workforce: A Qualitative Evaluation of Trained Lay Trauma Counsellors’ Experiences When Refugee Youth Disclose Suicidal Ideation	Sweden	Published research	Experiences of heath staff supporting youth refugees with suicidal ideation through TRT therapy.
2	Role of health and support staff in suicide prevention	2021	Ferguson, M et al.	Staff Perspectives of Safety Planning as a Suicide Prevention Intervention for People of Refugee and Asylum-Seeker Background.	Australia	Published research	Experiences of health staff in barriers and considerations of safety plan development for refugee and asylum seeker clients.
3	Suicide prevention training for staff supporting refugees and asylum seekers	2021	Procter, N et al.	An Evaluation of Suicide Prevention Education for People Working With Refugees and Asylum Seekers.	Australia	Published research	Evaluation of a 2 day refugee and asylum seeker suicide prevention training
4	Role of health and support staff in suicide prevention	2020	Byrow et al.	Tell Your Story': a randomized controlled trial of an online intervention to reduce mental health stigma and increase help-seeking in refugee men with posttraumatic stress	Australia	Published research	Online mental health stigma reduction program specifically designed for refugees.
5	Screening practices, interpreters and trauma informed care	2020	Posselt, Baker, Deans, & Procter.	Fostering mental health and well-being among workers who support refugees and asylum seekers in the Australian context	Australia	Published research	Staff mental health when working with refugees and asylum seekers
6	Suicide prevention training for staff supporting refugees and asylum seekers	2019	Procter, N.	Preventing Refugee and Asylum Seeker Suicide	Australia	Published research	Suicide prevention training and intervention
7	Suicide prevention training for staff supporting refugees and asylum seekers	2019	Momartin, McGrath, Nemorin, Coello, & Bibby.	STARTTS in schools: Integrating evaluation and practice to support students from refugee backgrounds.	Australia	Published research	School based mental health and suicide prevention program
8	Role of health and support staff in suicide prevention	2018	N. Procter, M. Kenny, H. Eaton, & C. Grech.	Lethal hopelessness: Understanding and responding to asylum seeker distress and mental deterioration	Australia	Published research	Trauma informed practical assistance and support for asylum seekers living in the community
9	Suicide prevention training for staff supporting refugees and asylum seekers	2018	Colucci, Jorm, Kelly, & Minas.	Suicide first aid guidelines for assisting persons from immigrant or refugee background: a Delphi study	Australia	Published research	Identifying and supporting suicidality
10	Understanding of mental health and suicide by refugees, asylum seekers	2017	Hynie, M.	The Social Determinants of Refugee Mental Health in the Post-Migration Context: A Critical Review	Canada	Critical review	Effectiveness of mental health interventions
11	Understanding of mental health and suicide by refugees, asylum seekers	2016	Barghadouch et al.	Refugee children have fewer contacts to psychiatric healthcare services: an analysis of a subset of refugee children compared to Danish-born peers	Denmark	Published research	Barriers to accessing mental health care for refugee children
12	Screening practices, interpreters and trauma informed care	2015	Shannon et al.	Beyond Stigma: Barriers to Discussing Mental Health in Refugee Populations	USA	Published research	Conversation barriers in mental health for refugees
13	Screening practices, interpreters and trauma informed care	2015	Talbot, N., Pahlevan, B., & Boyles, J.	We cannot talk if we do not feel free	UK	Grey literature	Role of interpreters in clinical practice
14	Screening practices, interpreters and trauma informed care	2008	Rosenberg et al.	Through interpreters' eyes: comparing roles of professional and family interpreters.	Canada	Published research	Role of interpreters in clinical practice

## Results

### Search results

Of the academic databases searched, 447 search results were returned. Of the grey literature searched, The Australian Bureau of Statistics, Australian Human Rights Commission, New South Wales Government and The United Nations High Commissioner for Refugees (UNHCR) contained data and information regarding refugee and asylum seeker suicide that supported understanding of suicide and suicide prevention in both international Australian contexts, however was not training focused and excluded from this rapid literature review.

### Literature results

Of the search results, the content within the literature relating to a) refugee and asylum seeker specific suicide prevention training, and b) the effectiveness of refugee and asylum seeker suicide prevention training for health and support staff was limited. The researchers however identified a small number of publications that outlined the type of information and content for consideration in the development of refugee specific suicide prevention training.

The literature results showed potential benefit of a specific refugee and asylum seeker suicide prevention training if the training content included expert knowledge, clinical skills including screening practices, and perspectives of the lived experience of refugee and asylum seekers.

#### A. Inclusion of expert knowledge

The inclusion of expert knowledge may support the usefulness of refugee and asylum seeker suicide prevention training. A training program for support staff working with refugees and asylum seekers in the community led by researchers from The University of South Australia’s Mental Health and Suicide Prevention Research Group suggest that the effectiveness of the suicide prevention training program was due to the inclusion of expert knowledge, evidence and lived experience of refugee and asylum seeker suicidality in its development [2].

#### B. Clinical knowledge to improve screening

In addition to expert knowledge, the inclusion of clinical elements in suicide prevention training for health and support staff may increase staff ability to identify suicide risk in refugee and asylum seekers clients through knowledge of screening practices. Research from the New South Wales Service for the Treatment and Rehabilitation of Torture and Trauma Survivors (STARTTS) surrounding mental health and suicide prevention training for school staff found the inclusion of clinical content in suicide prevention training can help to increase suicide prevention screening in support staff working with refugee and asylum seeker populations [26].

Although clinical knowledge is not a prerequisite of many support roles, a level of knowledge in clinical practices for mental health and suicide may increase the confidence of the support workers in their ability to screen for and address mental illness and suicidality in their refugee and asylum seeker clients [26].

Suicide prevention training that includes information about mental health screening practices and techniques can improve screening rates and identification of suicidal risk. A 2015 US study exploring mental health screening feasibility and training of refugee health coordinators found staff who had received higher levels of mental health training were more likely to screen for war trauma, torture and other mental health symptoms [27]. The study used a 28 question survey to determine the feasibility of mental health screening for newly arriving refugee clients. The study found higher levels of staff training was related to increased mental health screening of refugee clients and increased clinical or service referrals [27].

Inclusion of examples of screening techniques and practices may be a beneficial inclusion in suicide prevention training for health and support staff working with refugees and asylum seekers. A 2018 Delphi study by Colucci et al. [28], investigating mental health and suicide first-aid guidelines in Australia for assisting persons from immigrant or refugee backgrounds, incorporated a range of behaviours and verbal phrases that may indicate the presence of mental illness or suicidality in refugees and asylum seekers into mental health first aid training guidelines. The study highlighted that the recognition of warning signs for mental illness and suicidality in refugees is different compared to recognition of warning signs in the wider Australian population, and that this knowledge is important to undertake screening and support of refugees and asylum seekers who may be experiencing suicidal ideation [28]. Colucci et al. [28] highlights the importance of training to provide health and support staff with the knowledge and skills to identify the difference in clinical presentation of psychological distress, and suicide warning signs in refugees and asylum seekers.

#### C. Case studies

The use of case studies and role play within suicide prevention training for health staff working with refugees and asylum seekers may improve health staff confidence and ability to respond to suicidal distress. A 2021 study by Procter et al. saw improved participant competence and confidence after completing a specialised training course may be due to the use of case studies and examples to help participants understanding of suicidal psychology and behaviour [29].

#### D. Role play

The benefit of role play in training was also identified by Löfving Gupta et al. [30] amongst a range of other training elements. The Swedish study examined the experience of Teaching Recovery Techniques (TRT) trained counsellors in regards to knowledge, feelings of safety and competence to respond to suicidal distress in unaccompanied refugee youth clients, and asked counsellors what training elements would be required for them to appropriately respond to and manage suicidal risk in refugee clients. Training elements that were identified by counsellors as important were the need for repeat training sessions to support ongoing practice, training content regarding safety planning in practice, information about safety protocol including defining the boundaries between professional support when suicidal ideation is disclosed, and roleplay and examples of conversations to respond to suicidal ideation [30]. Similarly, a 2021 study by Ferguson et al. highlighted the benefit of repeat training session and the inclusion of safety planning suicide prevention training [31].

#### E. Cultural responsiveness

Refugee and asylum seeker suicide prevention training should consider the inclusion of content that provides health and support staff with the skills to communicate in a culturally responsive way. Existing literature highlights the differences in mental health and suicide literacy between different cultural groups. Colucci et al. [28] explored the importance of asking refugees and asylum seekers about their understanding of mental health and suicide and how it is perceived in their home country as a key component in responding to suicide risk. Colucci et al. [28] suggests training that teaches skills in cultural appropriateness, impacts or benefits of using interpreting services, and the benefit of management of suicide risk through referrals to cultural or spiritual services and groups in conjunction with mainstream medical and psychological services may support effective suicide prevention intervention [28].

Suicide prevention training that teaches health and support staff the skills to communicate in a culturally appropriate way, that also meet the literacy needs and understanding of mental health of refugee and asylum seeker clients is paramount. Ferguson et al. [31] highlighted the importance of staff skills to overcome language and communication barriers with refugee and asylum seeker clients to respond to suicide risk. Ferguson et al. recognises the need for health and support staff to have the skills and creativity to move beyond a written safety plan to manage suicide distress [31].

#### F. Co-design training

The value of co-designing suicide prevention training with refugee and asylum seekers has been recognised by Procter et al. 2021. The research paper highlights the collaboration with asylum seekers with lived experience of attempted suicide and/or witnessing suicidal behaviour in held detention and/or in the community to develop a suicide prevention training package that is culturally responsive and reflects the experiences of refugees and asylum seekers [29].

## Discussion

Existing literature in the space of refugee and asylum seeker mental health and suicide primarily focuses on psychosocial, economic, cultural and individual factors that impact on mental health and suicidality, with few publications examining what mental health and suicide prevention interventions work best in preventing suicidal distress in refugees and asylum seekers. Of the literature focusing on training as an intervention, there are limited publications demonstrating the effectiveness of suicide prevention training for health and support staff working with refugees and asylum seekers.

Of the identified literature in this review, there was some evidence to support the effectiveness of training to improve health and support staff knowledge, competence and ability to identify and respond to suicide which in turn may prevent suicide deaths. The University of South Australia’s Mental Health and Suicide Prevention Research Group designed and delivered a targeted two-day suicide prevention education program to government and non-government staff working with refugees and asylum seekers in the fields of nursing, social work, case work and volunteering. Findings from the study suggested the training led to significant improvement in refugee support workers’ confidence, attitude and competence to identify, manage and reduce suicide [2].

Continuing to explore the effectiveness of suicide prevention training on health and support staff working with refugees and asylum seekers, Procter et al. (2021) evaluated the outcomes of a specially designed suicide prevention training program specifically designed for and delivered to 400 staff members, volunteers and students from Australian non-government organisations who provide mental health support and case management to refugee and asylum seekers. The research study found an increase in competence and confidence, and change in attitude, of participants following undertaking a two-day specialised training program [29].

### Screening practices, interpreters and trauma informed care

#### Screening practices

Screening practices are a critical component in identifying suicide risk and should be considered as the primary step in identifying suicide risk in refugees and asylum seekers. Shannon et al. [27] found that during mental health and suicide prevention screening practices for refugee and asylum seekers, health staff reported difficulty in asking refugees and asylum seekers about their past experiences with trauma and torture. Shannon et al. (2015) reported that clinicians felt uncomfortable to cause refugees to re-live their experiences when asking about their history of past trauma, limiting ability for refugees and asylum seekers to disclose trauma related mental health concerns [27]. For support workers who may or may not be clinically trained, asking refugees or asylum seekers about their experiences of trauma may produce an emotional response that refugee support workers may not have suitable skills and confidence to respond to [27]. In addition, findings from the study by Shannon et al. [27] on screening practices used by staff found that staff who possessed higher levels of mental health training, demonstrated higher levels of screening practices and referrals for refugee and asylum seeker clients. This study is one such example highlighting the potential benefit of refugee and asylum seeker specific suicide prevention training for health and support staff.

#### Trauma informed approaches

Procter et al. 2018 identifies the risk of not using trauma informed approaches when working with and screening refugee and asylum seeker consumers. Health staff and support workers must consider the risk and benefit of asking refugee and asylum seeker consumers to recall on past traumtic events to prevent further stress. In favour, Procter et al. 2018 encourages health and support staff to include screening practices that gain an understanding of ‘what has happened to this person’ as opposed to ‘what is wrong with this person’ (Procter et al. 2018, pg. 451). With the above mentioned studies, development of suicide prevention training should consider the inclusion of content that not only improves the knowledge and competence of support staff to identify and respond to suicide risk, but should also provide guidance in screening practices and trauma informed care.

#### Use of interpreters

Even with strong screening practices, the ability of refugees and asylum seekers to communicate traumatic experiences and their emotional state may be either supported or hindered by the use of an interpreting service and individual understanding of mental illness and suicide [32]. For some refugees and asylum seekers, the use of interpreters can be beneficial in screening for mental illness and suicidality by creating a level of trust through use of native language [33]. Similarly, the understanding of mental illness and suicide by refugees and asylum seekers may impact the type of support that can be provided by health and support staff [9, 34]. There are significant differences in acceptability and understanding of suicide between refugee and asylum seeker groups, of which is impacted by cultural and social influences, and personal beliefs. These differences in the mental health literacy of refugee and asylum seekers, in conjunction with the cultural and social recognition (or lack of) of mental health, mental illness, and suicide can impact disclosure of suicidality to health staff and support workers [9, 34].

### Role of suicide prevention training for health and support staff

Suicidality in refugees and asylum seekers is influenced by a range of complex personal, social and economic factors and experiences. The ability of health and support staff working with refugees and asylum seekers to identify and support suicidality may support an early intervention approach to suicide prevention. Due to the increased risk of mental illness and suicide in refugees and asylum seekers, it is important that health and support staff engaging with refugees and asylum seekers have the competence and confidence to identify and manage suicide risk.

The literature results included in the review highlight the potential for suicide prevention training to increase the knowledge, skills and confidence of health and support staff to identify and respond to suicide risk in refugees and asylum seekers, providing the training includes core components that reflect the existing evidence. Evaluation of the training model explored in Procter et al. 2021, suggests the inclusion of role plays, or ability to observe others respond to suicidal distress, the inclusion of practical skills and tools such as safety planning, and the varying states of distress that a refugee or asylum seeker experiencing suicidal distress may encounter were training components that provided most benefit to the training participants [29].

Specific suicide prevention training for health and support staff working with refugees and asylum seekers can provide the specific skills required to support refugees and asylum seekers experiencing suicidality or psychological distress [29]. With reference to the studies cited in this rapid literature review, specific refugee and asylum seeker suicide prevention training should consider incorporating content that:

i. increases the knowledge and confidence of training participants to assist refugees and asylum seekers experiencing psychological distress [29].
ii. develops the competency of participants to respond to and manage suicide risk in refugees and asylum seekers [29, 30].
iii. addresses the importance of trauma informed and culturally appropriate approaches when working with refugees and asylum seekers [9, 28, 31, 34].
iv. is informed by the lived experience of refugees and asylum seekers [29].

Improving the knowledge and confidence of health and support staff working with refugee and asylum seekers through suicide prevention training may improve screening practices to support an increase in early identification of mental illness and suicidality. Increase in staff knowledge, confidence and competence as the result of suicide prevention training may lead to increased referrals to appropriate services, again supporting an early intervention approach to refugee and asylum seeker suicide prevention.

### Limitations

This rapid literature review identified a small number of published research items. Expanding the literature search to include research relating to mental health, mental illness, and distress may capture other published literature. Similarly, extending the search terms to include medical staff or staff and support workers from other disciplines again may return a higher number of literature results with valuable information. The method used to screen articles using title and abstract to determine potential inclusion of literature instead of reading full text may have also captured other information not included in this review. Finally, the inclusion of English only publications may have also impacted literature results.

### Recommendations

Given the increased risk of suicide for refugees and asylum seekers, further research exploring what factors are the biggest contributors to suicidality for refugees and asylum seekers would strengthen academic knowledge in best practice suicide prevention, and provide a foundation to inform key components for inclusion in refugee and asylum seeker specific suicide prevention training. In addition, further research to determine the efficacy of refugee and asylum seeker specific suicide prevention training to support an early intervention approach to suicide prevention would contribute valuable knowledge to this emerging field of research. Expanding literature search to include literature relating to suicide prevention and cultural considerations for different cultural groups may also inform content for inclusion in refugee and asylum seeker suicide prevention training.

## Conclusion

There are a number of social, economic and individual factors contributing to increased suicide risk in refugees and asylum seekers. Health and support staff working with refugee and asylum seeker consumers are well positioned to identify mental illness and suicidality in refugee clients. Specially designed refugee and asylum seeker specific suicide prevention training for health and support staff working with refugees and asylum seekers has the potential to improve the competence and confidence of health and support staff to effectively identify and respond to suicide risk in refugee and asylum seekers.

## Data Availability

Not applicable

## Appendices

### Appendix 1: Search strategy

Databases searched:

i. BioMed Central (Part of Springer Nature)
ii. EBSCOhost Academic Search Ultimate
iii. EBSCOhost- Sociology Source Ultimate
iv. ELSEVIER Clinical Key
v. ELSEVIER Science Direct
vi. Informit, ProQuest
vii. PubMed Central
viii. SAGE journals
ix. Taylor & Francis Online
x. Wiley Online Library.

*Limit to:* Full text only, English only,

*Date of Publication:* Published between January 2020 and January 2022.

*Document type:* Journal articles, books, reports, reviews, official publications, literature reviews.

### Appendix 2: Search terms

**Table:**

**Theme**	**Search terms**
Refugee and asylum seeker suicide prevention training for health workers	(suicide* AND prevent* AND training AND health) AND (worker OR staff OR employee) AND (refugee OR asylum)

### Appendix 3: Example search

**Table:**

Query	Sort By	Filters	Search Details	Results	Time
(suicide* AND prevent* AND training AND health) AND (worker OR staff OR employee) AND (refugee OR asylum)		from 2000/1/1 - 2022/1/1	("suicid*"[All Fields] AND "prevent*"[All Fields] AND ("education"[MeSH Subheading] OR "education"[All Fields] OR "training"[All Fields] OR "education"[MeSH Terms] OR "train"[All Fields] OR "train s"[All Fields] OR "trained"[All Fields] OR "training s"[All Fields] OR "trainings"[All Fields] OR "trains"[All Fields]) AND ("health"[MeSH Terms] OR "health"[All Fields] OR "health s"[All Fields] OR "healthful"[All Fields] OR "healthfulness"[All Fields] OR "healths"[All Fields]) AND ("occupational groups"[MeSH Terms] OR ("occupational"[All Fields] AND "groups"[All Fields]) OR "occupational groups"[All Fields] OR "worker"[All Fields] OR "workers"[All Fields] OR "worker s"[All Fields] OR ("staff"[All Fields] OR "staff s"[All Fields] OR "staffs"[All Fields]) OR ("employee s"[All Fields] OR "occupational groups"[MeSH Terms] OR ("occupational"[All Fields] AND "groups"[All Fields]) OR "occupational groups"[All Fields] OR "employee"[All Fields] OR "employees"[All Fields])) AND ("refugee s"[All Fields] OR "refugees"[MeSH Terms] OR "refugees"[All Fields] OR "refugee"[All Fields] OR ("asylum"[All Fields] OR "asylum s"[All Fields] OR "asylums"[All Fields]))) AND (2000/1/1:2022/1/1[pdat])	3	23:55:55
